# The Sand Fly Salivary Protein Lufaxin Inhibits the Early Steps of the Alternative Pathway of Complement by Direct Binding to the Proconvertase C3b-B

**DOI:** 10.3389/fimmu.2017.01065

**Published:** 2017-08-31

**Authors:** Antonio F. Mendes-Sousa, Vladimir Fazito do Vale, Naylene C. S. Silva, Anderson B. Guimaraes-Costa, Marcos H. Pereira, Mauricio R. V. Sant’Anna, Fabiano Oliveira, Shaden Kamhawi, José M. C. Ribeiro, John F. Andersen, Jesus G. Valenzuela, Ricardo N. Araujo

**Affiliations:** ^1^Physiology of Hematophagous Insects Laboratory, Department of Parasitology, Universidade Federal de Minas Gerais, Belo Horizonte, Minas Gerais, Brazil; ^2^Campus Senador Helvídio Nunes de Barros, Universidade Federal do Piauí, Picos, Piauí, Brazil; ^3^Laboratory of Simuliids and Onchocerciasis, Instituto Oswaldo Cruz, Fiocruz, Rio de Janeiro, Rio de Janeiro, Brazil; ^4^Vector Molecular Biology Section, Laboratory of Malaria and Vector Research, National Institute of Allergy and Infectious Diseases, National Institutes of Health, Rockville, MD, United States; ^5^Instituto Nacional de Ciência e Tecnologia em Entomologia Molecular, Rio de Janeiro, Rio de Janeiro, Brazil; ^6^Vector Biology Section, Laboratory of Malaria and Vector Research, National Institute of Allergy and Infectious Diseases, National Institutes of Health, Rockville, MD, United States

**Keywords:** sand fly, saliva, Lufaxin, complement system inhibition, alternative pathway

## Abstract

Saliva of the blood feeding sand fly *Lutzomyia longipalpis* was previously shown to inhibit the alternative pathway (AP) of the complement system. Here, we have identified Lufaxin, a protein component in saliva, as the inhibitor of the AP. Lufaxin inhibited the deposition of C3b, Bb, Properdin, C5b, and C9b on agarose-coated plates in a dose-dependent manner. It also inhibited the activation of factor B in normal serum, but had no effect on the components of the membrane attack complex. Surface plasmon resonance (SPR) experiments demonstrated that Lufaxin stabilizes the C3b-B proconvertase complex when passed over a C3b surface in combination with factor B. Lufaxin was also shown to inhibit the activation of factor B by factor D in a reconstituted C3b-B, but did not inhibit the activation of C3 by reconstituted C3b-Bb. Proconvertase stabilization does not require the presence of divalent cations, but addition of Ni^2+^ increases the stability of complexes formed on SPR surfaces. Stabilization of the C3b-B complex to prevent C3 convertase formation (C3b-Bb formation) is a novel mechanism that differs from previously described strategies used by other organisms to inhibit the AP of the host complement system.

## Introduction

Sand flies are dipteran plant feeders which females must feed on vertebrate blood in order to maturate their eggs. To be able to blood feed on a vertebrate host, the sand fly salivary secretion contains proteins, peptides, and small molecules aimed at modulating hemostatic responses of the host, including coagulation, vasoconstriction, and platelet aggregation, as well as inflammation and immune responses (1). In saliva of *Lutzomyia longipalpis*, the main vector of visceral leishmaniasis in the Americas, different molecules with antihemostatic properties have been characterized, such as the vasodilator Maxadilan (2, 3), the anticoagulant Lufaxin (4), the endonuclease Lundep (5), and, recently, the inhibitor of the classical pathway of the complement system SALO (6).

The complement system is part of the innate immune response that is responsible for the opsonization of cell surfaces, generation of potent anaphylatoxins, and direct killing of invasive pathogens and altered self-cells (7). Moreover, it is involved in antigen presentation and lymphocyte activation, through facilitation of the adaptive immune response (8, 9). Composed of more than 30 plasma and membrane proteins, the complement system can be triggered through three pathways, the classical (CP), lectin (LP), and alternative (AP). Once activated, these pathways converge at the C3 convertase and continue in a common pathway leading to formation of the membrane attack complex (MAC) that causes cell death (10). The complement components are also responsible for non-lethal effects such as cell activation and proliferation, resistance to subsequent complement lysis and either resistance to or induction of apoptosis (11).

The AP is primarily activated by spontaneous hydrolysis of the C3 component, forming soluble C3-H_2_O. This form binds factor B, that is then cleaved by factor D, generating C3-H_2_O-Bb. This soluble convertase cleaves more C3 molecules to C3b that go on to recognize surface hydroxyl or amino groups and covalently bind to them (12, 13). Factor B binds to deposited C3b forming the AP C3 proconvertase (C3b-B) that is then cleaved by factor D, forming the active C3 convertase of the AP (C3b-Bb). This complex activates more C3 molecules but has a short half-life of about 90 s, requiring properdin for stabilization (14). Activation of C3 leads to self-amplification and generation of the C5-convertase C3b-Bb-C3b, directing the formation of the MAC (7). As it is being constantly activated in the blood, the AP is a crucial mechanism of immune surveillance. Moreover, it has been shown to play a critical role in amplification of both the CP and LP and is responsible for 80% of the C5a and MAC generated by complement pathways (15).

*Lutzomyia longipalpis* saliva inhibits both the CP and the AP (16, 17). This inhibition is important for successful blood feeding in that it can diminish the inflammatory response at the bite site and protect the insect’s midgut from deleterious effect of the MAC (18–20). SALO, the *L. longipalpis* salivary inhibitor of the CP, was recently described as being an 11 kDa protein that acts on the first steps of the cascade (6). In this paper, we show that Lufaxin, a known *L. longipalpis* salivary anticoagulant (4) and a candidate vaccine for leishmaniasis (21), is the inhibitor of the AP in unfractionated saliva. We also demonstrate that Lufaxin binds to the C3b-B complex and inhibits activation of factor B and consequently the formation of the C3 convertase, a unique mode of action not seen in other organisms.

## Materials and Methods

### Ethics

All animal procedures were reviewed and approved by the National Institute of Allergy and Infectious Diseases (NIAID) Animal Care and Use Committee under protocol LMVR4E and handled in accordance to the Guide for the Care and Use of Laboratory Animals and with the NIH OACU ARAC guidelines and also approved by Ethics Committee in Animal Experimentation (CETEA) of Universidade Federal de Minas Gerais (UFMG) under Protocol no. 87/2011.

### Production of Sand Fly Recombinant Salivary Proteins

Sand fly transcripts coding for Lufaxin and 11 other salivary proteins were cloned into the VR2001-TOPO vector as described before (21, 22). Recombinant proteins were produced in Leidos Biomedical Research PEL facility by transfecting HEK 293 F cells with the VR2001-TOPO DNA plasmids coding for the different sand fly salivary proteins and incubated for 72 h. The supernatant was concentrated and further purified by HPLC (NGC Chromatography system, Bio-Rad Laboratories, Inc.) using a HiTrap chelating HP column (GE Healthcare) charged with Ni_2_SO_4_. Imidazole was removed from fractions containing Lufaxin by washing with PBS using a 5,000, 10,000, or 30,000 MWCO Amicon filter (Millipore). Purified Lufaxin was analyzed by NuPage 4–12% gels (4) and stored at −80°C until use. Aliquots did not undergo more than three freeze-thaw cycles.

### Detection of Anti-Complement Activity

Twelve recombinant salivary proteins from *L. longipalpis* were tested on standardized AP-mediated hemolysis assays in order to detect anti-complement activity as previously described (23). The rabbit erythrocytes were acquired from CompTech or collected by venous puncture from a rabbit kept on the animal facility of UFMG. Before the experiments, 500 µl of rabbit blood were washed three times in 5 ml of Mg-EGTA solution (1 mM HEPES, 30 mM NaCl, 10 mM EGTA, 7 mM MgCl2, 3% glucose, and 0.02% gelatin, pH 7.4) as described in Ref. (16). The erythrocyte concentration was adjusted to 1 × 10^8^ cells/ml. All the experiments were performed at pH 7.4, unless specified. Briefly, in 1.5 ml microcentrifuge tubes, 25 µl of normal human serum (NHS, CompTech) diluted 1:20 in Mg-EGTA buffer (1 mM HEPES, 30 mM NaCl, 10 mM EGTA, 7 mM MgCl_2_, 3% glucose, and 0.02% gelatin, pH 7.4) were mixed with 12.5 µl of PBS containing 1 µg of each recombinant protein. Then, 25 µl of Mg-EGTA containing 2.5 × 10^6^ rabbit red blood cells were mixed and the tubes incubated for 30 min at 37°C for complement activation. Final concentration of NHS in 62.5 µl of buffer was 2%. After incubation, 250 µl of cold PBS were added and the tubes rapidly centrifuged. Two hundred microliters of the supernatants were transferred to microplates and read at 415 nm. Tubes incubated without any recombinant protein were used as positive controls and tubes without NHS were used as negative controls. Tubes containing red blood cells but without NHS and recombinant proteins were combined with 250 µl of distilled water and were used to obtain total hemolysis. The assays were performed in duplicate, and in every test the mean of negative control was subtracted from the mean of the other results. The results were then transformed in percentage of lysis, considering tubes treated with distilled water as 100% of hemolysis.

In some experiments, AP-mediated hemolytic assays were performed with different concentrations of recombinant Lufaxin or LJS169 using Mg-EGTA buffer at pH 7.4 and pH 8.15 [pH in the intestinal lumen of *L. longipalpis* right after blood meal (24)]. Similar assays were also carried out using properdin-depleted serum (CompTech) diluted 1:2 in Mg-EGTA buffer (pH 7.4) and final concentrations of Lufaxin ranging from 0 to 200 nM.

In order to investigate if Lufaxin inhibits classical pathway, CP-mediated hemolytic assays were performed using IgG-sensitized sheep red blood cells (CompTech) as described by Ferreira et al. (6). Possible inhibition of LP by Lufaxin and LJS169 was investigated as described in the study of Mendes-Sousa et al. (23) through detection of the deposition of C4b component on *Saccharomyces cerevisiae*’s mannan-coated microplates using anti-C4 polyclonal antibodies (CompTech).

### Blocking of Lufaxin Inhibition with Antiserum

Balb/c mice were injected three times every 2 weeks, intradermally in the ear with 5 µg of recombinant Lufaxin mixed (1:1 volume) with Magic™ Mouse Adjuvant (Creative Diagnostics) as recommended by the manufacturer. Fifteen days after the last inoculation, blood was collected to obtain the Lufaxin antiserum. The ability of Lufaxin antiserum to recognize the native and recombinant Lufaxin was checked by Western blot as previously described (4). Lufaxin (500 ng) and SGH (five pairs of *L. longipalpis* glands) were run in a NuPAGE 4–12% gel (ThermoFisher Scientific) and transferred to nitrocellulose membranes (ThermoFisher Scientific). Membranes were blocked with 5% non-fat milk solution overnight, incubated with Lufaxin antiserum (1:1,000 dilution) or mice pre-immune serum (control; 1:1,000 dilution), followed by anti-mouse secondary antibodies (Promega), and developed by addition of Western Blue^®^ stabilized substrate for alkaline phosphatase (Promega).

Rabbit erythrocytes (5 × 10^6^) with NHS (1:20) and samples were incubated at 37°C for 30 min, and the percentage of hemolysis was quantified. Erythrocytes incubated with distilled water were considered as 100% of hemolysis. Results were expressed as mean ± SEM of at least three independent experiments. Lufaxin (100 nM) or *L. longipalpis* salivary gland homogenate (SGH) was incubated at 37°C for 10 min with varying dilutions of anti-Lufaxin serum prior to the AP-hemolytic assays. The Lufaxin-serum or SGH/serum mixture was then used in hemolytic assay as described above.

### Detection of Complement Component Deposition on Agarose-Coated Plates

ELISA plate (Corning Inc.) wells were filled with 100 µl of 0.1% agarose solution and dried completely by overnight incubation at 37°C. Then, 100 µl of HMEBN buffer (5 mM HEPES, 7 mM MgCl_2_, 10 mM EGTA, 5 mg/ml BSA, and 140 mM NaCl, pH 7.4) containing 20% NHS and different concentrations of Lufaxin or LJS169 were added to the wells and the plate incubated at 37°C for 30 min under agitation. After two washes, the wells were incubated for 30 min with antibodies against each specific complement components produced in goat [C3, factor B, properdin, C5, or C9 (CompTech)] diluted in 10 mM HEPES and 140 mM NaCl solution. After two more washes, the wells were treated with anti-goat peroxidase-conjugated antibodies (Sigma-Aldrich) and then developed with 200 µl of developing buffer (50 mM Na_3_C_6_H_5_O_7_, 50 mM Na_2_HPO_4_, 1 mg/ml o-phenylenediamine (Sigma-Aldrich), and 0.075% H_2_O_2_, pH 5.0). The plate was read at 450 nm at kinetic mode (one read every 30 s) at 37°C for 10 min. Wells without serum and without recombinant protein were used as negative and positive control, respectively. The assays were performed in triplicate. The mean of negative controls was subtracted from the mean of other results and then transformed in percentage of deposition considering the positive control as 100% deposition.

### Detection of Complement Activation by Western Blotting

To detect activation of complement components in the presence of Lufaxin, Western blotting assays were performed as described in the study of Mendes-Sousa et al. (23). First, AP-mediated hemolytic assays were done as described above in the presence of Lufaxin, LJS169, or PBS. After different times of incubation (0, 30, and 60 min), an aliquot of the supernatant of each tube was collected, mixed with denaturing sample buffer, and loaded onto SDS gels. Purified factor B (10 ng), Bb (10 ng), and C3a (5 ng) (CompTech) were loaded on the SDS gels as controls.

In the assays for detection of C3 activation, an aliquot with diluted NHS without inhibitor and red blood cells was also loaded onto the gel. After transfer to nitrocelullose membranes (Bio-Rad Laboratories, Inc.), the membranes were blocked for 2 h with 0.05% Tween 20 and 10% dried non-fat milk in PBS under agitation and then incubated with goat anti-factor B or rabbit anti-C3a antibodies (CompTech) diluted 1:1,000 in 0.05% Tween 20 and 1% BSA in PBS for 1 h. After washing three times with 0.05% Tween 20 in PBS, the membranes were treated with anti-goat or anti-rabbit peroxidase-conjugated antibodies (Sigma-Aldrich) diluted 1:3,000 in the same buffer of primary antibodies for 1 h. Visualization of the proteins was possible after treating the membranes with the peroxidase substrate diaminobenzidine kit (Vector Laboratories).

### Hemolytic Assays to Detect Inhibition of MAC Assembly

To determine if Lufaxin acts at MAC formation, AP-mediated hemolytic assays were performed with some modifications. Primarily, in 1.5 ml microcentrifuge tubes, 25 µl of C6-depleted serum (CompTech) diluted 1:20 in Mg-EGTA buffer were mixed with 12.5 µl of PBS containing recombinant Lufaxin or LJS169 and 25 µl of rabbit red blood cells and incubated at 37°C for 15 min. The final concentration of the recombinant proteins was 75 nM. Tubes without recombinant proteins (only PBS) were used as negative controls. After incubation, the tubes were centrifuged (1 min, 1,700 *g*) and the supernatant discarded. Erythrocytes were resuspended in a mixture of 12.5 µl PBS plus 50 µl of NHS diluted 1:40 in GHB-EDTA buffer (5 mM HEPES, 145 mM NaCl, 10 mM EDTA, and 0.1% gelatin, pH 7.4) and incubated at 37°C for 30 min. After this step, the assay followed as described previously.

For detection of inhibition after the C6 component (MAC assembly), the assay was performed without recombinant protein during the first incubation, and the erythrocytes were resuspended in a mixture of 12.5 µl of PBS containing Lufaxin or LJS169 and 50 µl of NHS diluted 1:40 in GHB-EDTA buffer. Detection of hemolysis was evaluated as described above.

### Enzymatic Assay of Factor D

In order to check if Lufaxin affects factor D activity, enzymatic assays based on Kam et al. (25) were performed. Purified factor D (6 µg/ml) (CompTech), Lufaxin (0.1 µg), and 0.1 mM aldrithiol (Sigma-Aldrich) were combined in 0.1 M HEPES, 0.05 M NaCl, and 10% DMSO (pH 7.4) buffer (100 µl final volume) in 1.5 µl microcentrifuge tubes. After homogenization, the content of the tubes was transferred to ELISA microplates and 100 µl of 2 mM Z-1-Lys-SBzL substrate (Sigma-Aldrich) diluted in the same buffer was added to the wells. The plate was immediately read at the kinetic mode at 37°C for 30 min at 324 nm. Tubes without Lufaxin and factor D were used as positive and negative controls, respectively. Tubes with only Lufaxin, aldrithiol, and the substrate were also used to check if Lufaxin have any effect over the substrate.

### Detection of Direct Binding of Complement Components to Lufaxin Immobilized on Microplates

ELISA plates (Corning Inc.) were incubated overnight at 4°C with 50 µl of coating buffer (35 mM Na_2_CO_3_, 15 mM NaHCO_3_, pH 9.6) containing 0.5 µM of purified C3b, properdin, factor B, Bb, or factor D (CompTech). Wells incubated with 1% BSA in coating buffer were used as negative controls. Following a 1 h blocking with 1% BSA in PBS, the wells were incubated with 120 nM of Lufaxin in 50 µl of PBS for 30 min under agitation. The plate was washed twice with 0.05% Tween 20 in PBS and then treated with mouse anti-Lufaxin serum diluted 1:500 in blocking buffer (1% BSA in PBS). After two more washes, the wells received 50 µl of blocking buffer containing alkaline phosphatase-conjugated anti-mouse antibody (Sigma-Aldrich) diluted 1:10,000 for 30 min. The plate was washed and the reaction was completed by adding 100 µl of p-nitrophenyl phosphate substrate for alkaline phosphatase (Sigma-Aldrich). After 20 min of incubation at 37°C, the plate was read at 450 nm.

### Detection of Direct Binding Lufaxin to Complement Components by Surface Plasmon Resonance (SPR)

C3b was diluted in 10 mM sodium acetate buffer (pH 5.0) and immobilized on CM5 sensor chips (GE Healthcare) by the amine-coupling method. Assays were conducted at 25°C on a Biacore T100 instrument (GE Healthcare) by passing different combinations of complement factors and Lufaxin over the C3b immobilized surface. The buffer for these experiments (HBS-N) contained 10 mM HEPES, pH 7.4, 150 mM NaCl. In most cases, either MgCl_2_ or NiCl_2_ was added to the buffer to a concentration of 2 mM.

### Assays for Inhibition of C3 and Factor B Cleavage

To assay the ability of Lufaxin to inhibit the activation of C3, the C3b-Bb complex was formed in solution by incubating 200 nM C3b, 100 nM factor B, and 50 nM factor D in HBS-N buffer containing 2 mM MgCl_2_ for 2 min at room temperature. After stopping the formation of activated complex by the addition of EDTA (5 mM), purified C3 was added at various concentrations in the presence or absence of Lufaxin (1 µM), and the mixtures were incubated for 20 min at room temperature before the addition of SDS-PAGE sample preparation buffer. Inhibition of factor B activation was evaluated by incubating 200 nM C3b, 100 nM factor B, and 50 nM factor D in the presence or absence of 1 µM Lufaxin in HBS-N containing 2 mM MgCl_2_ for 30 min. The reactions were stopped by adding EDTA to a concentration of 5 mM. In both types of experiments, the reaction products were separated by SDS-PAGE and blotted to nitrocellulose. To detect C3 cleavage, blots were incubated with rabbit anti-C3a, and to detect factor B cleavage they were incubated with goat anti-factor B, followed by incubation with the appropriate alkaline phosphatase conjugate antibody. The blots were then developed using Western Blue alkaline phosphatase substrate reagent (Promega).

### Data Analysis

Inhibition of samples was analyzed using GraphPad Prism 5.0 software. All assays were carried out in duplicate (hemolytic assays) or triplicate (deposition assays) with at least three biological repetitions. Mean of groups was statistically compared using ANOVA followed by Tukey’s test using 95% as significant level (*p* < 0.05).

## Results

### Lufaxin Is the Alternative Pathway (AP) Inhibitor from the Saliva of the Sand Fly *L. longipalpis*

**Figure 1:**
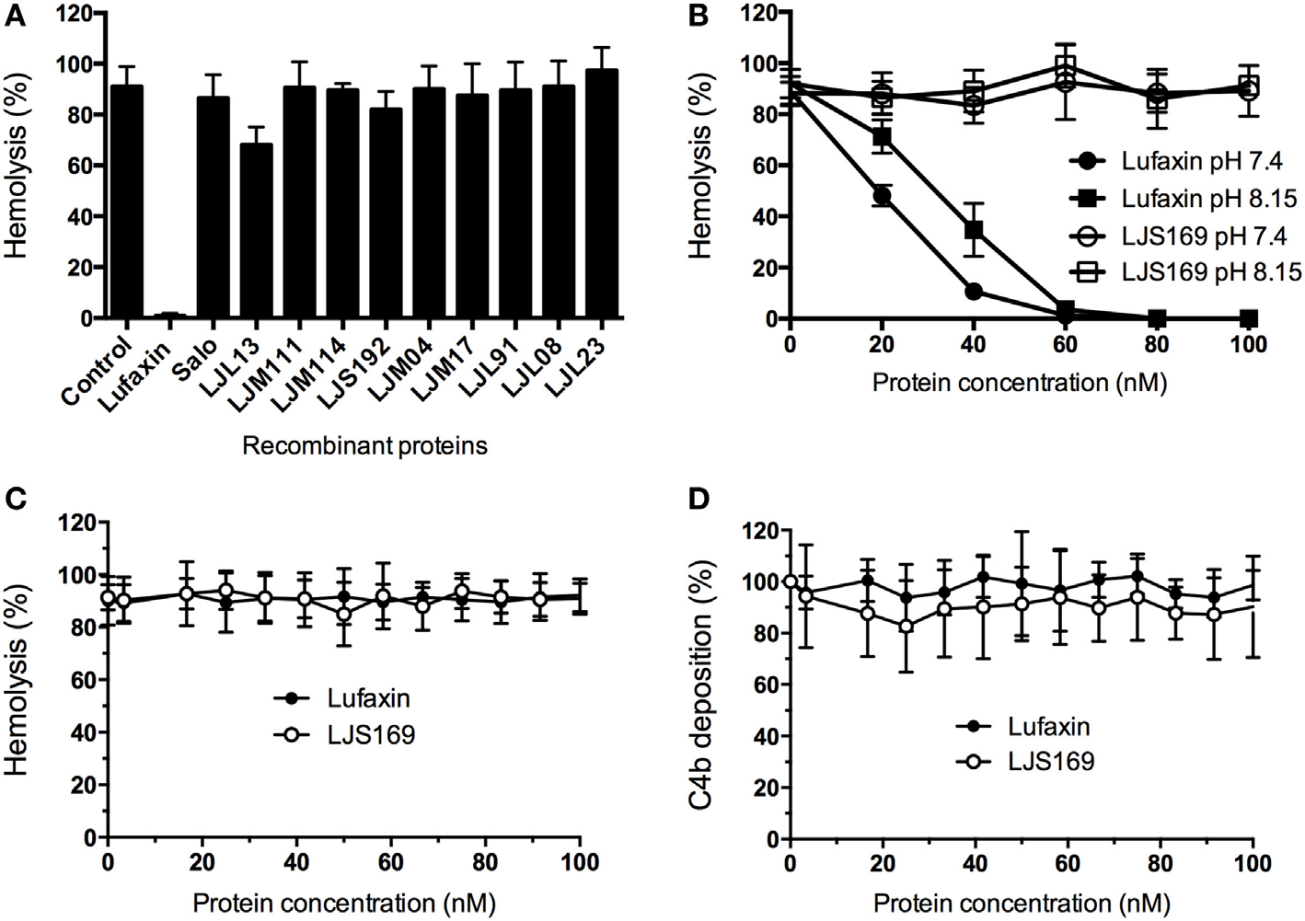
Lufaxin is the alternative pathway (AP) salivary inhibitor of *Lutzomyia longipalpis*. **(A)** Recombinant salivary proteins expressed in HEK 293-F cells were tested on the AP-mediated hemolysis assay using rabbit red blood cells and normal human serum (NHS). Only Lufaxin caused significant inhibition of lysis (*p* < *0.001*, ANOVA + Tukey test). **(B)** Lufaxin caused inhibition of hemolysis in a dose-dependent manner at pH 7.4 or pH 8.15. LJS169 was used as negative control as it did not present anti-complement activity. **(C)** Lufaxin and LJS169 were tested in the classical pathway hemolytic assay with NHS and antibody-sensitized sheep erythrocytes. Neither Lufaxin nor LJS169 inhibited hemolysis. **(D)** Mannan-coated microplates were used to activate the lectin pathway. Lufaxin or LJS169 was added together with NHS and incubated at 37°C. The deposition of C4 was measured using anti-C4 antibodies and no effect was seen either for Lufaxin or LJS169.

To identify the AP-complement inhibitor in *L. longipalpis* saliva, 12 recombinant salivary proteins, chosen from among the most abundant molecules (26), were tested in AP hemolytic assays. Only one sand fly salivary recombinant protein of 32.5 kDa, Lufaxin, produced a complete reduction in AP erythrocyte lysis (Figure 1A). Lufaxin inhibited AP erythrocyte lysis in a dose-dependent manner at host physiological pH (7.4) and sand fly midgut pH (8.15) (24) in comparison to the control protein LJS169, another sand fly recombinant salivary protein (Figure 1B). Statistical significance was observed at Lufaxin concentrations of 40 nM or above (*p* < 0.0001), confirming its inhibitory effect on the AP.

The possible activity of Lufaxin against other complement pathways was also examined. The CP-mediated hemolytic assay (Figure 1C) and LP-mediated activation (Figure 1D) were not inhibited by Lufaxin or the control protein LJS169.

### Anti-Lufaxin Antibodies Block AP-Inhibition

**Figure 2:**
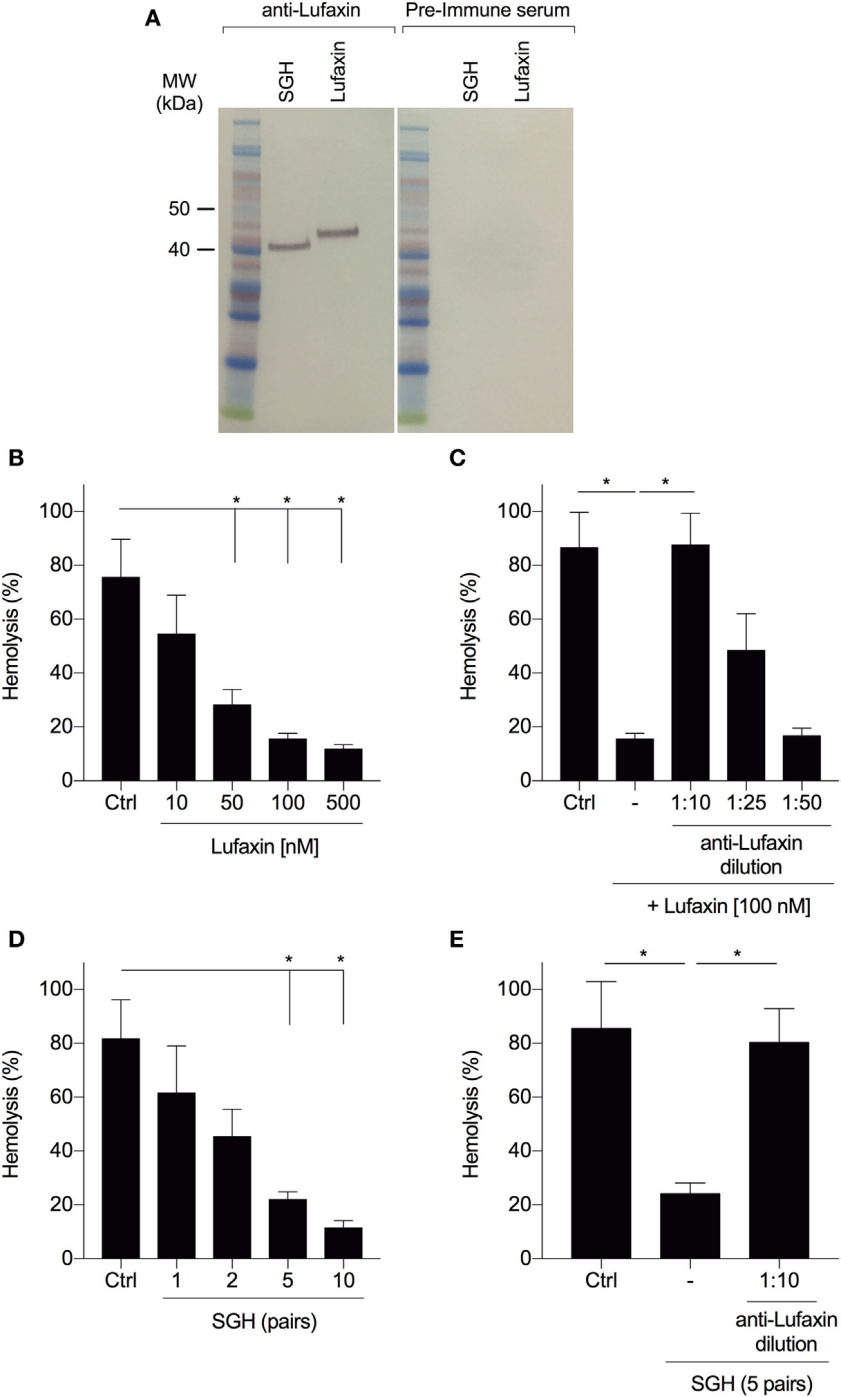
Antibodies to Lufaxin block the inhibitory effect of Lufaxin and sand fly saliva on the AP of complement. **(A)** Anti-Lufaxin serum recognizes both recombinant Lufaxin (500 ng) and native Lufaxin on *Lutzomyia longipalpis* salivary gland homogenate (SGH – five pairs). Lufaxin or SGH was loaded in a NuPAGE 4–12% gel, followed by Western blotting using anti-Lufaxin polyclonal antibody. Mice preimmune serum was used as negative control. **(B)** AP hemolytic assays were carried on with different concentrations of Lufaxin. **(C)** Lufaxin (100 nM) was preincubated with different dilutions of anti-Lufaxin serum for 10 min and tested on AP-hemolytic assays. **(D)** AP-hemolysis assays were carried out with different amounts of *L. longipalpis* pairs of SGH. **(E)** *L. longipalpis* SGH (5 pairs) were preincubated with anti-Lufaxin serum (1:10) for 10 min and tested on AP-hemolytic assays. **(B–E)** 5 × 10^6^ rabbit erythrocytes were incubated with normal human serum (1:20) and samples at 37°C for 30 min, and the percentage of hemolysis was quantified. PBS was used in negative controls (Ctrl). Erythrocytes incubated with distilled water were considered as 100% of hemolysis. Results are expressed as mean ± SEM of at least three independent experiments.

We tested whether neutralization of Lufaxin by antibodies would prevent blockage of AP-mediated rabbit erythrocyte hemolysis by Lufaxin or sand fly saliva. Anti-Lufaxin serum produced in mice was able to recognize both the recombinant and native Lufaxin (Figure 2A). Recombinant Lufaxin inhibited AP activation in a dose-dependent manner (Figure 2B), and addition of anti-Lufaxin serum to recombinant Lufaxin partially rescued complement activation at a dilution of 1:25, and completely rescued activation at a 1:10 dilution (Figure 2C). In order to investigate if Lufaxin is the only AP inhibitor in *L. longipalpis* saliva, we tested the effect of anti-Lufaxin serum on *L. longipalpis* SGH in the AP hemolysis assay. First, we confirmed that SGH also inhibited AP activation in a dose-dependent manner (Figure 2D) and next that addition of anti-Lufaxin serum at a dilution of 1:10 completely rescued AP complement activation (Figure 2E). These results indicate that Lufaxin accounts for most or all AP-inhibitory activity in *L. longipalpis* saliva.

### Lufaxin Blocks the Early Steps of the AP Complement Cascade

**Figure 3:**
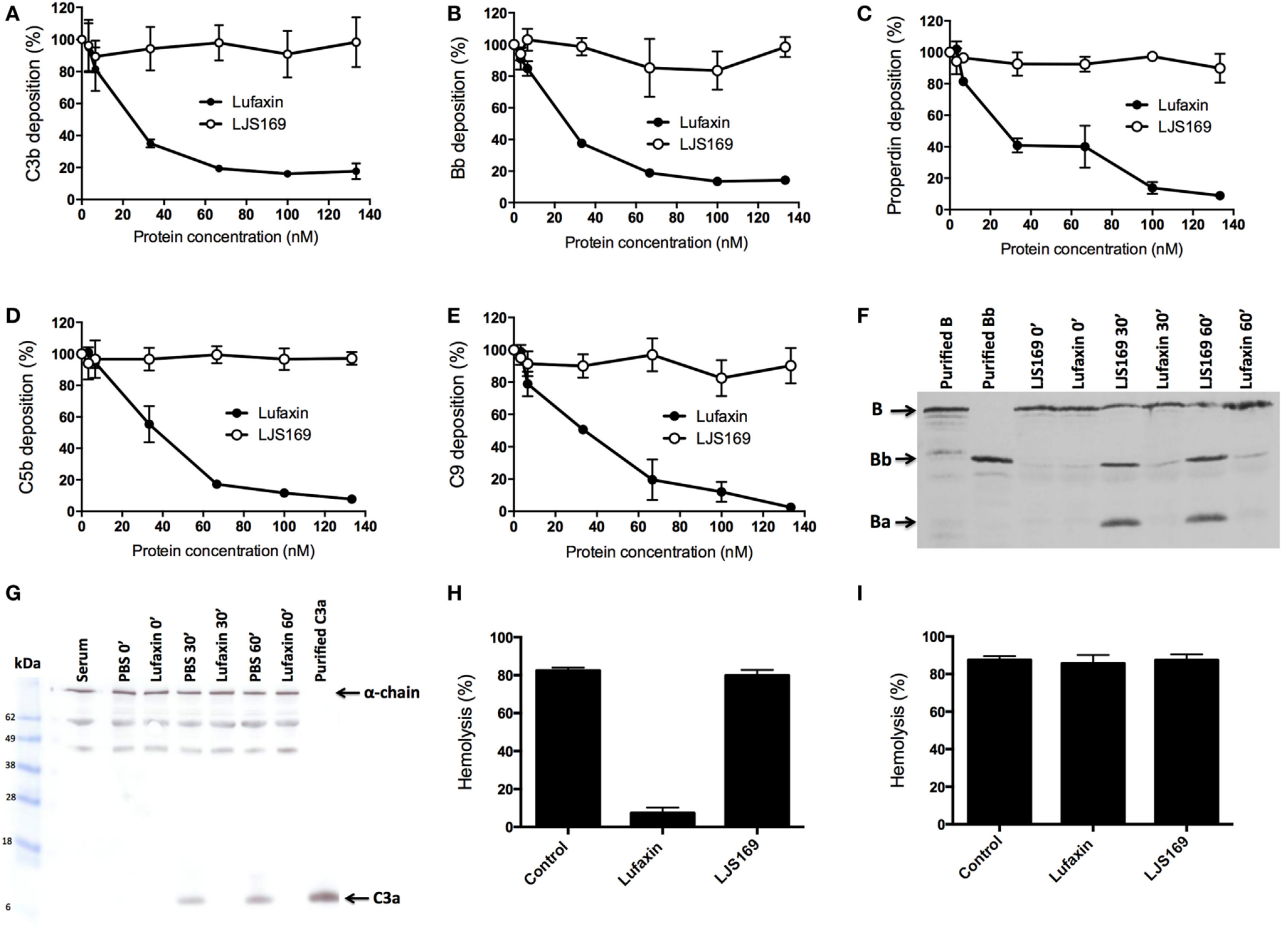
Lufaxin acts exclusively at the early steps of the alternative pathway (AP) of the complement system. **(A–E)** Normal human serum (NHS) was added to agarose-coated microplates together with Lufaxin or LJS169 (as negative control) and incubated at 37°C for AP activation. Deposition of C3b **(A)**, Bb **(B)**, properdin **(C)**, C5b **(D)**, and C9 **(E)** was assessed using specific antibodies. **(F,G)** AP-mediated hemolysis assays were performed in the presence of Lufaxin, LJS169 or PBS. At different times of incubation (0′, 30′, and 60′), supernatants were collected and submitted to SDS-PAGE. Proteins were transferred to nitrocellulose membranes and then incubated with anti-factor B **(F)** or anti-C3a antibodies **(G)**. A control aliquot with diluted NHS without inhibitor and red blood cells (Serum) was also loaded onto the gel. **(H,I)** Effect of Lufaxin or LJS169 on formation of the membrane attack complex. Rabbit erythrocytes were first incubated with C6-depleted serum at 37°C, and after incubation, the cells were centrifuged and the supernatant discarded. The cells were then resuspended with NHS in EDTA buffer (blocking the initial steps of the cascade) and incubated again. Lufaxin or LJS169 was added in the first **(H)** or second **(I)** incubation.

Agarose-coated microplates were used to measure the deposition of complement components after activation of the AP. In the presence of Lufaxin, deposition of C3b, the first activated component of the AP, was strongly inhibited in a dose-dependent manner (Figure 3A). This correlates with the inhibition of subsequent components of the complement cascade (Bb, properdin, C5b, and C9) by Lufaxin (Figures 3B–E). The negative control (LJS169) showed no effect on the deposition of the tested components (Figures 3A–E).

Inhibition of complement activation by Lufaxin was further confirmed by Western blotting of supernatants from the AP-hemolysis assay using anti-factor B and anti-C3a polyclonal antibodies in the presence of Lufaxin or LJS169. Lufaxin blocked factor B cleavage, since no bands (or very weak bands) corresponding to factor Bb or Ba were detected on immunoblots at 30 or 60 min after AP complement initiation as compared to treatment with LJS169 (Figure 3F). Similarly, when Lufaxin was added to tubes with serum and red blood cells, a band corresponding to C3a was not observed, indicating that it inhibited the activation of C3, even after 60 min at 37°C (Figure 3G). These results strongly suggest that Lufaxin prevents the formation of C3b and Bb, the components of the AP C3-convertase complex.

Hemolysis assays were then performed to determine if Lufaxin acts exclusively at the early steps of the activation cascade or may act on any MAC component. Hemolytic assays were carried out using C6-depleted serum and red blood cells, and, after removal of the supernatant, MAC formation was elicited by addition of NHS diluted in buffer containing EDTA to prevent the formation of additional C3 convertase complexes. When Lufaxin was added with the C6-depleted serum, hemolysis was significantly reduced (*p* < 0.0001), suggesting that Lufaxin impairs C3 convertase formation (Figure 3H). The strong hemolysis seen in tubes without inhibitor, or with the control recombinant LJS169, indicates that the C6-depleted serum was active (Figure 3H). When Lufaxin was added together with the NHS, after C3 convertase assembly, no effect on MAC formation was observed (*p* = 0.7657) (Figure 3I), providing further evidence that Lufaxin acts exclusively on the early steps of the AP, where C3b and Bb are produced.

### Lufaxin Binds to the C3b-B Complex and Blocks Factor B Activation

**Figure 4:**
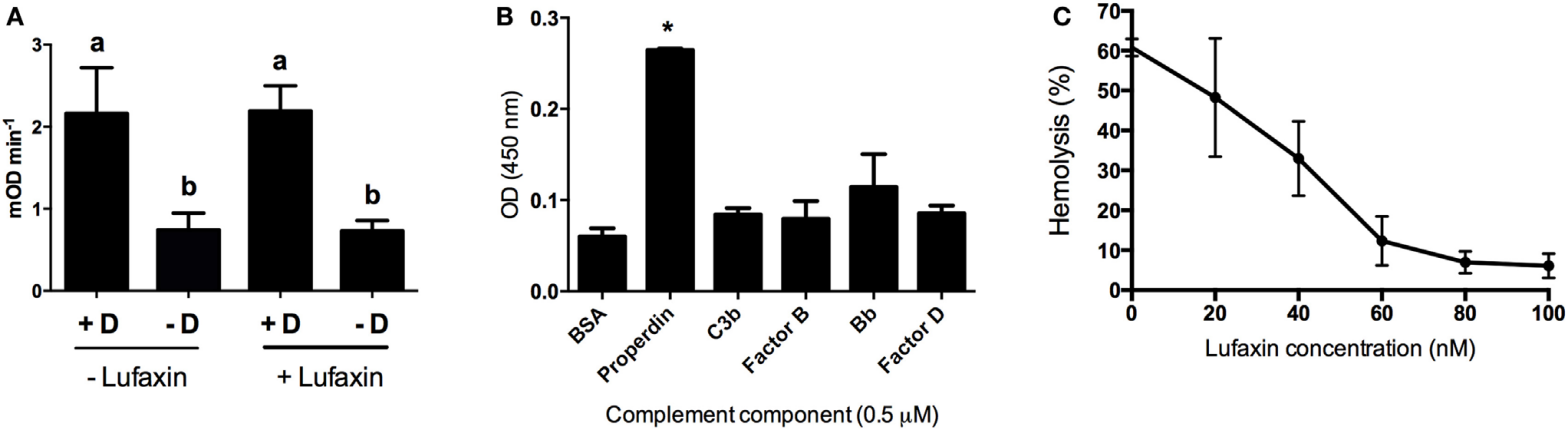
Effect of Lufaxin on factor D activity and on the direct binding of complement components. **(A)** Effect of Lufaxin on factor D activity. Purified factor D (+D) was mixed with specific substrate Z-1-Lys-SBzL in the presence or absence of Lufaxin and incubated at 37°C. Tubes without factor D (−D) were used as controls. Different letters indicate statistical difference between groups (*p* < 0.05). **(B)** ELISA plates were sensitized with 0.5 µM of purified properdin, C3b, factor B, Bb, factor D, or 1% BSA followed by incubation with 120 nM of Lufaxin. Mouse anti-Lufaxin serum was used to detect significant binding. Asterisk indicates statistical difference (*p* < 0.01) from controls (BSA). **(C)** Different concentrations of Lufaxin were carried on AP-hemolytic assays using properdin depleted serum.

To determine how Lufaxin impairs C3-convertase formation, we first tested whether it is a direct inhibitor of factor D, the serine protease that cleaves factor B in the AP pathway. Lufaxin, purified factor D, and aldrithiol were mixed and added to factor D substrate Z-1-Lys-SBzL. Similar levels of substrate cleavage were seen in tubes containing factor D with or without Lufaxin, indicating that Lufaxin does not inhibit the enzymatic activity of factor D (Figure 4A).

As Lufaxin does not act on factor D, we then checked which AP complement components may be affected by Lufaxin. ELISA plates were coated with different AP complement components followed by addition of Lufaxin which was able to bind significantly to properdin (*p* < 0.001). Lufaxin was not able to bind significantly (*p* > 0.05) to C3b, factor B, Bb, and factor D when they were alone on the plate surface (Figure 4B).

The role of Lufaxin binding to properdin was assessed by hemolytic assays using properdin-depleted sera. Lufaxin maintained its AP inhibitory activity even in the absence of properdin (Figure 4C), suggesting properdin binding is not essential for AP inhibition.

**Figure 5:**
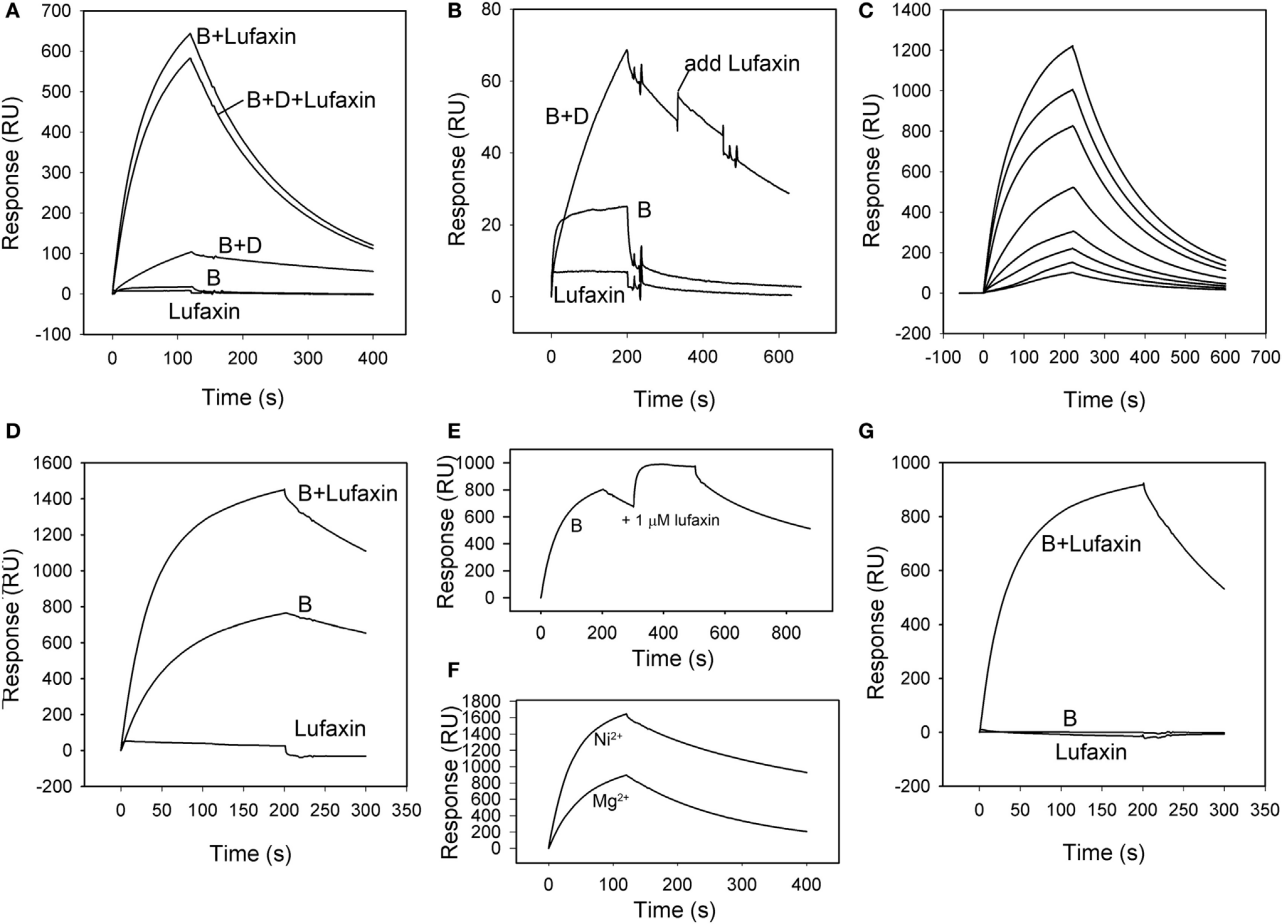
Surface plasmon resonance analysis of the effect of Lufaxin on C3b-B and C3b-Bb assembly in the presence of Mg^2+^ or Ni^2+^. **(A)** Lufaxin (MW = 32.5 kDa) injected in combination with factor B or factor B plus factor D stabilizes the formation of proconvertase and convertase complexes on an immobilized C3b surface (3,100 RU). B + Lufaxin = 54 nM factor B + 1 µM Lufaxin, B + D + Lufaxin = 54 nM factor B + 20 nM factor D + 1 µM Lufaxin, B + D = 54 nM factor B + 20 nM factor D, B = 54 nM factor B, and Lufaxin = 1 µM Lufaxin. **(B)** Lufaxin does not bind to a pre-assembled C3b-Bb complex (C3b surface, 3,000 RU immobilized). Injection mixtures are labeled as in panel **(A)**. “Add Lufaxin” indicates an injection of 1 µM Lufaxin made after assembly of C3b-Bb [B + D]. Trace shows little or no increase in resonance response due to Lufaxin binding. **(C)** Concentration-dependence of Lufaxin stabilization of C3b-B (C3b surface, 3,500 RU immobilized). Increasing concentrations of Lufaxin were injected over a C3b surface along with 54 nM factor B. Traces in order of increasing amplitude indicate Lufaxin concentrations of 15.6, 31.2, 62.5, 125, 250, 500, and 1,000 nM. **(D)** Effect of Lufaxin on formation of C3b-B in the presence of Ni^2+^ (C3b surface, 3,000 RU immobilized). Lufaxin, factor B, and factor B + Lufaxin were injected over a C3b surface in the presence of 2 mM NiCl_2_. The plots are labeled as in panel **(A)**. **(E)** Binding of Lufaxin to a preformed C3b-B complex in the presence of Ni^2+^ (C3b surface, 3,000 RU immobilized). The C3b-B complex was formed on the same surface as in panel **(D)** (line B), and during the dissociation phase, 1 µM Lufaxin was injected. **(F)** Comparison of C3b-B stabilization by Lufaxin in the presence of Mg^2+^ and Ni^2+^. Factor B + Lufaxin [as in panel **(A)**] was injected over the same C3b surface using HBS-N containing MgCl_2_ or NiCl_2_ as the sample and running buffer. **(G)** Stabilization of C3b-B by Lufaxin in the absence of divalent cations (C3b surface, 3,150 RU immobilized). Injections were made in HBS-N buffer alone. Traces are labeled as in panel **(A)**.

The ability of Lufaxin to bind the active C3 convertase C3b-Bb, or the proconvertase C3b-B, was evaluated using SPR. In these experiments, factor D, factor B, and Lufaxin were run in different combinations over surfaces of immobilized C3b in the presence of Mg^2+^. Factor B bound detectably to immobilized C3b, and stability of the complex was significantly increased by adding factor D to the mobile phase (Figures 5A,B), indicating the formation of C3b-Bb. Lufaxin alone did not bind C3b (Figures 5A,B), but when it was added to factor B, or to the factor B-factor D mixture prior to injection, a large increase in the stability of the complex formed on immobilized C3b was observed (Figure 5A). Under conditions of constant factor B concentration, complex formation was found to depend on the concentration of Lufaxin (Figure 5C). These results indicate that Lufaxin binds to the C3b-B complex and possibly to the C3b-Bb complex. To distinguish these possibilities, the C3b-Bb complex was formed by injection of a mixture of factor B and factor D over a C3b surface (Figure 5B) and, after formation of the complex, Lufaxin was injected alone while the complex slowly dissociated. No significant Lufaxin binding was observed in this case, suggesting that the inhibitor binds only to the C3b-B complex and not to C3b-Bb (Figure 5B). The fact that complex stabilization by Lufaxin was essentially equivalent in the presence and absence of factor D suggests that the inhibitor stabilizes the C3b-B complex, while preventing activation of factor B by factor D (Figure 5A).

Magnesium ion binds at the metal ion-dependent adhesion site (MIDAS) of the VWA domain of factor B and is coordinated by the C-terminus of C3b (27, 28). Replacement of Mg^2+^ with Ni^2+^ stabilizes an open form of C3b-bound factor B that is susceptible to cleavage by factor D (29, 30). To gain insight into the mechanism of complex formation we examined the ion specificity of C3b-B-Lufaxin formation using SPR. Binding of factor B to a C3b surface in the presence of Ni^2+^ was enhanced, and the addition of Lufaxin led to a further increase in the amplitude of the sensorgram (Figure 5D). However, fitting the dissociation phase data to a single exponential decay function indicated that the half-time for C3b-B-Lufaxin dissociation (~400 s) was of similar magnitude to that for C3b-B (~600 s) suggesting that Lufaxin binds to C3b-B, but does not provide the degree of stabilization in the presence of Ni^2+^ seen in the presence of Mg^2+^ (Figures 5A,F, *t*1/2 ~ 135 s). Injection of Lufaxin on the same surface after formation of the C3b-B complex (during the dissociation phase) showed an increase in binding to produce a total sensorgram amplitude that was similar to that seen with coincident injection of factor B and Lufaxin (Figure 5E, B + Lufaxin), again suggesting that Lufaxin binds to the complex but does not further stabilize the interaction of factor B with C3b in the presence of Ni^2+^. Comparison of assembly on the same C3b surface in Ni^2+^ and Mg^2+^ buffers revealed a higher level of C3b-B-Lufaxin complex formation in the presence of Ni^2+^ than Mg^2+^ (Figure 5F), but comparison of Figures 5A,D,E suggests that Lufaxin enhances the binding of factor B to the C3b surface in the presence of Mg^2+^, but not Ni^2+^. Perhaps, Lufaxin binds preferentially to the open form of C3b-bound factor B, thereby increasing its abundance in the presence of Mg^2+^ but not in the presence of Ni^2+^ where the open form is already favored. When divalent cations were removed completely (HBS-N buffer alone) neither factor B nor Lufaxin bound detectably to the C3b surface, while binding of the factor B-Lufaxin mixture was similar to that seen in the presence of Mg^2+^ (Figure 5G). This indicates that the presence of Mg^2+^ or Ni^2+^ in the MIDAS of the VDW domain of factor B is not essential for formation of a C3b-B-Lufaxin complex and suggests that Lufaxin may stabilize the open form of factor B independently of metal ions.

**Figure 6:**
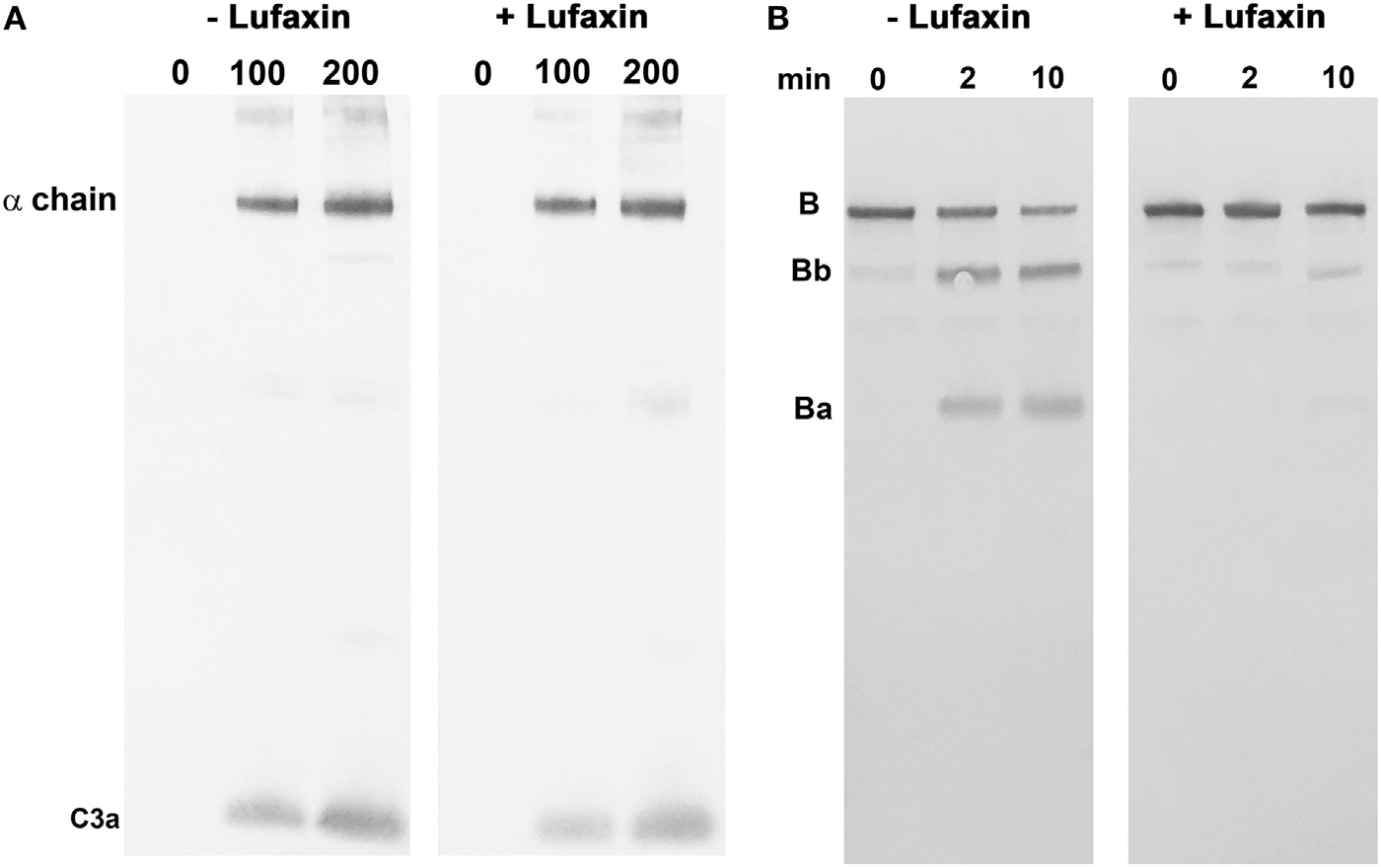
Effect of Lufaxin on the activation of factor B and C3 in reconstituted systems. **(A)** Cleavage of C3 by a preformed C3b-Bb complex. The complex was formed by incubation of a mixture of purified C3b, factor B, and factor D in the presence of Mg^2+^. After addition of EDTA, the complex was incubated with C3 and analyzed by immunoblotting using anti-C3a. The left panel shows incubations with 0, 100, and 200 nM C3 in the absence of Lufaxin, while the right panel shows the same set of concentrations in the presence of 1 µM Lufaxin. **(B)** Activation of factor B by factor D in a reconstituted C3b-B complex. Mixtures of C3b, factor B, and factor D were incubated in the presence and absence of 1 µM Lufaxin. Cleavage of factor B was evaluated by Western blot using anti-factor B. Cleavage is indicated by the appearance of Ba and Bb. The left panel shows results of incubations for 0, 2, and 10 min in the absence of Lufaxin, and the right panel shows the same incubations in the presence of Lufaxin.

The effect of Lufaxin on the enzymatic activity of the proconvertase and convertase complexes was tested using reconstituted systems. The C3b-Bb complex was assembled from soluble purified components by activating C3b-bound factor B with factor D in the absence of Lufaxin. The cleavage of C3 by the complex was then evaluated in the presence and absence of Lufaxin using Western blots to detect the release of C3a (Figure 6A). Under these conditions, Lufaxin did not inhibit C3 cleavage, which is consistent with SPR results showing a lack of binding to the C3b-Bb complex (Figure 6A). However, when C3b was mixed with factor B in the presence and absence of Lufaxin prior to the addition of factor D, cleavage of factor B, as indicated by the appearance of Bb on Western blots, was inhibited in the presence of Lufaxin, but not in its absence (Figure 6B). This result is also consistent with SPR data and supports the idea that Lufaxin binds to the C3b-B complex and inhibits the cleavage of factor B by factor D.

## Discussion

Saliva of *L. longipalpis* has previously been shown to inhibit both the classical and APs of human complement system, suggesting the presence of a single inhibitor of the common pathway, or of multiple anti-complement factors (16). An 11 kDa salivary protein, given the name SALO, was recently found to inhibit the classical pathway but not the AP, indicating that a second component must be present (6). In this study, we show that Lufaxin, a 32 kDa protein of 278 amino acids, first described as an inhibitor of coagulation factor Xa (4), is also an inhibitor of the AP. The protein shows no sequence similarity to any other known proteins with the exception of homologs found in the saliva of other sand fly species (31). Along with the previously described classical pathway inhibitor SALO, Lufaxin could potentially protect the feeding sand fly from the effects of anaphylatoxins and the tissue damaging effects of complement deposition and MAC assembly in the gut.

Among the arthropod AP-complement inhibitors, the inhibitory mechanism of Lufaxin is novel. Albicin, an AP inhibitor from saliva of the mosquito *Anopheles albimanus*, also targets the C3 convertase, but binds and inhibits C3b-Bb rather than C3b-B (23). The mechanism is also different from the AP inhibition elicited by other organisms such as ticks (32–34), mites (35), and bacteria (28).

Although the ligation of Lufaxin to properdin may play a role in the AP inhibition, the ligation is not the central mechanism of the Lufaxin AP inhibitory activity since it remained active in properdin depleted sera. Such fact distinguishes its mode of action from well-known AP-inhibition mechanisms involving properdin in saliva of ticks, which bind and remove properdin from the active AP-C3 convertase, reducing its half-life and accelerating its decay (32–34, 36). Inhibition of the AP C5-convertase by Lufaxin was not directly investigated. If present, this effect could increase the AP inhibition by Lufaxin.

Inhibition of the complement cascade at an early point, such as C3 convertase activation, would be beneficial to a blood feeder having a limited quantity of salivary secretion since the concentrations of these complexes are initially low. Additionally, blockage of the cascade before the production of anaphylatoxins (C3a and C5a) would limit any negative effect of these pro-inflammatory molecules while feeding. In the pool-feeding sand flies, this is apparently an adaptive mechanism, since they possess a limited amount of salivary proteins (less than 1 μg/gland) (17) and feed in a complex environment where local inflammatory responses are triggered by tissue damage caused by feeding (37–39).

The inhibition of the host complement system has also been shown to prevent complement-mediated damage to the gut of triatomine bugs (18) and mosquitoes (19), thereby improving the utilization of the blood meal. Considering that the pH in the midgut lumen of *L. longipalpis* becomes alkaline immediately after a blood meal [rising to 8.15 (24)] and that the complement system remains active in the midgut of hematophagous insects (18, 19), specially the AP (40), Lufaxin would need to be active in the physiological conditions where complement meets the midgut cells in order to have a protective effect. It is known that *L. longipalpis* ingests saliva with the contents of the feeding pool (41). Thus, Lufaxin starts to inhibit the AP at the bite site (on pH 7.4) and inhibition has to continue at the midgut pH (8.15). Such activity corroborates the strong and similar activity detected for Lufaxin in both pH 7.4 and 8.15.

In addition to blood feeding and midgut protection, Lufaxin may be also important for *Leishmania* transmission since it can protect recently injected promastigotes in host tissues. Both the alternative and classical pathways of the complement system are activated during *Leishmania* infection (42, 43). Although the parasite possesses its own mechanisms of evasion of the complement system, such as lipophosphoglycans and the metalloprotease gp63 (44), it is known that promastigotes are significantly sensitive to the complement system and must invade host cells rapidly in order to escape it.

Over the last two decades, several studies with rodent models have shown that protection against cutaneous or visceral leishmaniasis can be achieved by previous immunization with sand fly saliva or salivary proteins (45–49), acting as a transmission-blocking vaccine (TBV). Vaccination of dogs and mice with DNA plasmids encoding Lufaxin induced a delayed-type hypersensitivity (21, 50), that is the response mechanism underlying the adverse effects on *Leishmania* promastigotes (37). The induction of neutralizing antibodies able to reverse the anticoagulant and/or AP-inhibitory activity, at least in part, could increase the efficiency of Lufaxin as a TBV.

In addition to Lufaxin, SALO (the *L. longipalpis* CP inhibitor) also induced protective anti-*Leishmania* immunity in hamsters (47, 51). It is conceivable that a vaccine containing both proteins (Lufaxin and SALO) would potentially potentiate the effectiveness of a TBV.

## Ethics Statement

All animal procedures were reviewed and approved by the National Institute of Allergy and Infectious Diseases (NIAID) Animal Care and Use Committee under protocol LMVR4E and handled in accordance to the Guide for the Care and Use of Laboratory Animals and with the NIH OACU ARAC guidelines and also approved by Ethics Committee in Animal Experimentation (CETEA) of Universidade Federal de Minas Gerais under Protocol no. 87/2011.

## Author Contributions

MP, MS, FO, SK, JR, JA, JV, and RA conceived the study. AM-S, VV, NS, AG-C, JA, JV, and RA designed experiments. AM-S, VV, NS, AG-C, and JA performed experiments. All the authors contributed to analysis and interpretation of data. AM-S, VV, NS, AG-C, JA, JV, and RA wrote the paper. MP, MS, FO, SK, JR, JA, JV, and RA critically revised the paper. All the authors approved the manuscript.

## Conflict of Interest Statement

The authors declare that the research was conducted in the absence of any commercial or financial relationships that could be construed as a potential conflict of interest.
